# Water, Sanitation, and Hygiene (WASH) Practices and Outreach Services in Settlements for Rohingya Population in Cox’s Bazar, Bangladesh, 2018–2021

**DOI:** 10.3390/ijerph19159635

**Published:** 2022-08-05

**Authors:** ASG Faruque, Baharul Alam, Baitun Nahar, Irin Parvin, Ashok Kumar Barman, Soroar Hossain Khan, M Nasif Hossain, Yulia Widiati, ASM Mainul Hasan, Minjoon Kim, Martin Worth, Maya Vandenent, Tahmeed Ahmed

**Affiliations:** 1 Nutrition and Clinical Services Division, International Centre for Diarrhoeal Diseases Research, Bangladesh (icddr,b), Dhaka 1212, Bangladesh; 2 UNICEF Bangladesh, Cox’s Bazar Field Office, Cox’s Bazar 4700, Bangladesh; 3 UNICEF Bangladesh Country Office, Dhaka 1207, Bangladesh; 4 Office of Executive Director, International Centre for Diarrhoeal Diseases Research, Bangladesh (icddr,b), Dhaka 1212, Bangladesh

**Keywords:** WASH, case management, emergency crisis settings, diarrheal disease, disease surveillance, Rohingya refugee population

## Abstract

(1) Background: This study aimed to investigate the existing water, sanitation, and hygiene (WASH) policy and practice of the study population and strengthen the evidence base by documenting changes in the WASH policy and practice over 3 years of the Rohingya refugee humanitarian crisis, Cox’s Bazar, Bangladesh. (2) Methods: A cross-sectional surveillance design was followed; the sampling of the study population included the Rohingya refugee population and neighborhood host nationals who required hospitalization soon after seeking care and enrolled into the diarrheal disease surveillance in diarrhea-treatment centers. Throughout the study period of 3 years, a total of 4550 hospitalized individuals constituted the study participants. (3) Results: Among the hospitalized Rohingya refugee population; the use of public tap water increased significantly from 38.5% in year 1 to 91% in year 3. The use of deep tube well water significantly changed from 31.3% to 8.2%, and the use of shallow tube well water reduced significantly from 25.8% to 0.4%. Households using water seal latrine were 13.3% in year 1 and increased significantly to 31.7% in year 3. ORS consumption at home changed significantly from 61.5% in the first year to 82.1% in third year. Multivariable analysis demonstrated patients’ age groups at 5 to 14 years, and 15 years and more, drinking non-tube well water, soap use after using toilet, use of non-sanitary toilet facility, father’s and mother’s lack of schooling, and some and severe dehydration were significantly associated with the Rohingya refugee population enrolled into the diarrheal disease surveillance. (4) Conclusion: The findings indicate significant advances in WASH service delivery as well as outreach activities by aid agencies for the Rohingya refugee population living in settlements.

## 1. Introduction

Bangladesh is well-known to be vulnerable to natural disasters that occur almost every year [1]. In addition to these annual phenomena, over 17 weeks starting in late August 2017, the country experienced a rapid incursion of the Rohingya refugee population from the adjoining Rakhine state of Myanmar. Addressing this exceptional and substantial man-made humanitarian crisis, the Government of Bangladesh immediately responded by providing refuge to the Rohingya refugee population in diverse and widely distributed hilly and forested spaces of two sub-districts of Cox’s Bazar named Ukhia and Teknaf, situated in south-east Bangladesh [2].

Upon arrival, the distressed Rohingya refugee population was observed to be in immediate need of shelter, food, and health care services. Humanitarian agencies began providing housing, water, sanitation, and hygiene (WASH) services including latrines; water points as sources of water for drinking and domestic activities; bathing places with the provision of water, especially for women; and WASH supplies including soap and buckets for water collection and storage [3]. Due to the rapid onset of the refugee influx, some WASH services were not equitably allocated in the initial stages, leading to, for example, long waiting times and long travel distances for drinking water collection. Humanitarian response agencies made groundwater accessible through the construction of tube wells fitted with hand pumps to provide a potable water source. However, this construction took some time, and excess withdrawals of water from the shallow aquifer caused drying up of some of those tube wells. Moreover, due to a lack of an adequate quantity of safe water during these initial stages of the emergency, the Rohingya population, in the absence of choices, accessed drinking water from surrounding highly contaminated surface water sources, including paddy fields and irrigation canals. Many emergency tube wells were quickly installed; however, those were mostly shallow tube wells with a high risk of contamination [4]. Although a large number of emergency tube wells were installed, there was little control on quality standards, and in many cases, those were constructed without any prior planning and measurement of groundwater levels at the point of installation. Moreover, tube wells were not equally distributed, and many of these shallow tube wells were vulnerable to running out of water in the dry season (late November–April) in the absence of natural replenishing at the sub-soil level by rainwater. Over the centuries, vast areas of Cox’s Bazar are well-known for lacking adequate water sources because of the sharp falling of water levels making pumping water too difficult along with environmental mismanagement present in the forest and hilly topography that are restraining the resident population as well as the Rohingya population from accessing an adequate quantity of safe drinking water [5,6,7,8,9]. Additionally, being close to the sea, the groundwater from diverse sources has the potential for high salinity. There is also the risk of arsenic in the groundwater, as found in other parts of the country [10,11,12,13].

The quality of the drinking water from the very beginning was of high concern to the aid agencies. Water-quality surveillance indicated that a higher proportion of routinely monitored samples of water were highly contaminated with *E. coli* both at the collection point and household level, reflecting fecal contamination of water. Between 24 October and 12 November 2017, water samples from 624 sources and 1248 households were collected and tested. About two-thirds of water source samples and 93% of household water samples reported contamination with *E. coli*. Subsequent routine water-quality monitoring revealed that even if water is not contaminated at the source level, it is very likely to become contaminated at a household level due to inadequate knowledge and unsafe personal and domestic hygiene behaviors of the family members, for example, the storage of drinking water in uncovered containers such as jars and buckets. Boiling of drinking water was not often practiced due to lack of firewood. Water treatment practices, particularly the use of chlorine tablets by the Rohingya population, was lacking. According to one study, between February and September 2018, of the 893 water samples that were collected from tube wells (collection point), little over one-quarter had contamination with fecal coliforms, and 11% had observed *E. coli* pollution. However; regarding stored household water samples at the point of use, 74% were contaminated with fecal coliform, and 35% reported the presence of *E. coli* [14,15,16].

Soon after the arrival of the Rohingya refugee population, open defecation was reported to be a common practice in the camp areas due to the absence or insufficient numbers of latrines. Emergency pit latrines were hurriedly constructed to provide immediate access to sanitation facilities. With no clear standards or guidelines for latrine construction, many of these emergency latrines were of substandard design and construction, for example, with only a shallow pit, which quickly became filled and overflowed. Due to the crowded conditions in the camp areas, latrines were often built near one another as well as too close to shelters and water points. This caused higher risks of fecal pollution of surrounding sources of water. Because of absence of standardized protocols or facilities for the emptying of latrines and treatment of fecal sludge, many of the emergency latrines quickly became full and started overflowing, adding to the risk of contamination of shallow tube well water points. The absence of vacant land in the crowded camp areas delayed the establishment of appropriate fecal sludge-treatment plants and increased the risk of contamination of shallow tube wells, which heightened the threats of enteric disease outbreaks, including acute watery diarrhea (AWD) in general and cholera and shigellosis in particular [6,7,8,9,17,18].

Limited access of Rohingya population to water and soap within or outside latrines impeded appropriate personal hygiene practices after toilet use. Washing hands after handling children’s feces were practiced less frequently. Safe handling of water practices at household level were not widely followed by the Rohingya population living in temporary housing. Inadequate access to soap by the Rohingya refugee population caused low levels of handwashing practice, which was noted as a gap in the WASH sector response and that required the attention of humanitarian agencies to address the hygiene control measures [7,19].

The influx of large numbers of distressed and traumatized Rohingya refugees into a small, undeveloped area without adequate emergency services posed a major health risk, with disease outbreaks a certainty unless appropriate WASH, health, shelter, and other services were provided quickly. People in such a situation are vulnerable to rapid growth deterioration during or after any illness, particularly if they fail to meet their needs of any additional nutrient and calorie due to a breakdown in food supply and feeding practices that are associated with illness or convalescence. Public health researchers have indicated a high risk of outbreaks of waterborne diseases such as cholera or shigellosis in emergency and crisis settings. Providing access to quality water, sanitation, and hygiene services is essential in preventing diarrheal illnesses, consequent malnutrition, and deaths, particularly in young children [20,21,22,23,24].

In the early stage of the Rohingya refugee population’s arrival, whilst safe WASH services were being established, the challenges to adequate WASH services and associated behaviors, such as defecation outside the toilet, usage of unsafe water for drinking and household activities, low-level hygienic knowledge and behavior, overcrowding, and augmented mobility of the Rohingya population across the temporary housing areas, continued to pose a major risk of disease outbreak. Those prevailing scenarios triggered abrupt health risks in the Rohingya refugee population, including upsurges of AWD, cholera, and shigellosis [22,24].

During the influx, the International Centre for Diarrhoeal Disease Research, Bangladesh (icddr,b), and United Nations Children’s Fund (UNICEF) undertook a transitory situation analysis by visiting the Rohingya refugee population in shelters and talking to them and host-community individuals as well as field staff members of humanitarian agencies in two stated sub-districts of Cox’s Bazar. The appraisal predicted immediate warnings of large-scale upsurges of enteric diseases including cholera and shigellosis. Immediately, a partnership was developed concentrating on the (i) capacity building of diverse health workforce in managing along with referral of acute watery diarrhea (AWD) cases to in-patient and out-patient facilities, (ii) AWD case management in five UNICEF-established diarrhea-treatment centers (DTCs), and (iii) diarrheal disease surveillance in these DTCs. 

The health systems of the Government of Bangladesh, with practical cooperation from icddr,b and the coalition of international aid agencies along with international and national NGOs, launched large-scale oral cholera vaccine (OCV) campaigns from October 2017 to December 2018 as proactive measures to alleviate the likelihood of cholera outbreaks in the Rohingya refugee population who were living in large numbers in widely scattered settlements with the prevailing devastating scenario of limited access to well-organized WASH platforms [25,26,27].

The WASH practice of household members is a critical determinant of AWD, cholera, and shigellosis in the family and immediate neighborhood in settlements. It was hypothesized that due to the high vulnerability of the Rohingya population, they had a high likelihood of surges of AWD due to unsafe WASH practices of the family members. However, during the prevailing threats of cholera outbreaks, knowledge gaps related to safe hygiene knowledge and behaviors among the Rohingya refugee population and host population were identified. Reducing such information gaps would be critical to design effective strategies to prevent diarrhea, consequent malnutrition, and deaths, particularly among children, living in either the Rohingya refugee population settlements or nearby host communities. In the absence of monitoring and reporting systems, disease surveillance for AWD, cholera, and shigellosis was essential. 

Several pieces of research have indicated that measures for providing basic health services, including provision of adequate water, sanitation, and hygiene (WASH), are essential components in emergency crisis settings [28,29]. The WASH platform serves to prevent infectious diseases and their spread and sustain the dignity of vulnerable, displaced populations. This paper will update policymakers, public health experts, program implementation managers, the academic world, and wider stakeholders about the changing WASH strategy in humanitarian crisis settings in the settlements of the Rohingya population and neighborhood host communities in Cox’s Bazar. Such an apprising of this kind is expected to empower stakeholders to start necessary preparedness and response readiness to avoid disease outbreaks, particularly cholera or shigellosis outbreaks, from occurring and to combat them effectively when outbreaks may happen.

Additionally, rapid identification of any AWD cases is essential, particularly that of cholera or shigellosis, so that early warning and alert response systems (EWARS) can act promptly to control disease spread; ensuring these control strategies is more effective in avoiding deaths [30].

## 2. Materials and Methods

### 2.1. Study Design

A cross-sectional facility-based surveillance design was developed to monitor patients who were admitted because of acute diarrhea episodes at the icddr,b-operated network of diarrhea-treatment centers (DTCs). Although services were open to all, the Rohingya refugee population living in the nearby settlements and the host-community individuals from neighborhood locality were the prime service seekers. 

### 2.2. Study Site

**Figure 1:**
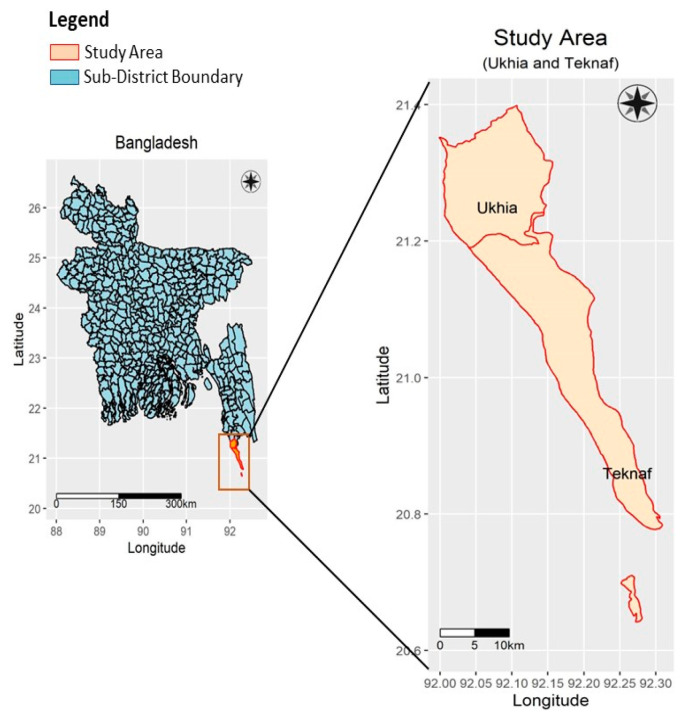
Geographical location of the study area.

The study was carried out in a network of DTCs situated in two sub-districts, namely Ukhia and Teknaf of Cox’s Bazar, Bangladesh. Figure 1 shows the geographical location of the study area.

Throughout year 1, five DTCs served the population. For year 2, only Teknaf and Leda DTCs continued to serve the patient population. Ultimately, since the beginning of year 3 to date, Leda DTC is the single DTC providing care to diarrhea-sick individuals from settlements and host communities. Details of the DTCs are shown in Table 1.

### 2.3. Study Population

**Figure 2:**
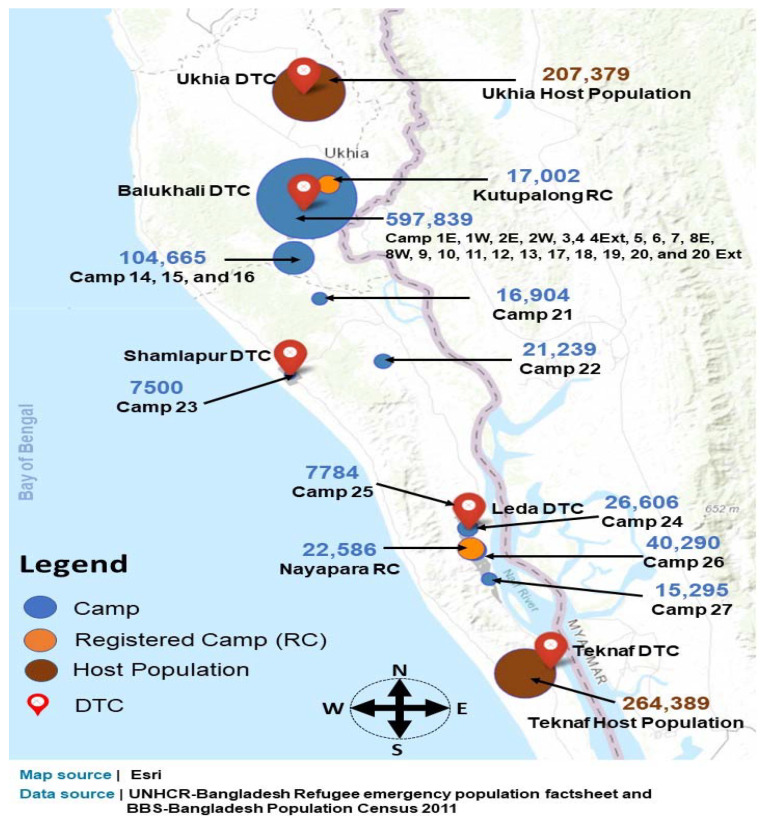
Rohingya refugee camps in Ukhia and Teknaf.

The study population was comprised of the Rohingya refugee population who are dwellers of the widely distributed emergency shelters and Bangladeshi nationals who are residents of surrounding host communities. Details of distribution of camps, the Rohingya population, host communities, and the host population along with the location of DTCs are shown in Figure 2.

According to the Bangladesh Government and the UNHCR joint report of 31 March 2022, the Rohingya refugee population has an average family size of five and a growth rate of 3.77%. The report indicated the Rohingya population’s age group: 51% are children between 0 and 17 years, 45% are adults between 18 and 59 years, older persons (60 years and more) comprise 4%, and persons with disabilities comprise 1%. In the adult age group (18–59), 19.6% is male and 24.3% female. In this group, the male-to-female ratio is 100:124. In the Rohingya children’s category (0–17 years), male-to-female ratio is 100:95. The host population reported a family size of 5.5 and a growth rate of 1.3%, whereas overall male-to-female ratio in the host population is 100:101. Moreover, 37% of the host population is aged 0–17 years, 55% is adult (18–59 years), and 8% of the individuals are older, namely 60 years and above. Nearly 2% of the host population has disabilities. In the adult (18–59 years) age group, 54% are males and 57% females in the host population. The male-to-female ratio in this age group is 100:101. The male-to-female ratio for the host-community children (0–17 years) is 100:96 [31,32].

The sampling of the study population of this cross-sectional DTC-based surveillance design included those from the Rohingya refugee population and neighborhood host nationals who required hospitalization soon after arrival and enrolled into the diarrheal disease surveillance. The large and unprecedented influx of the Rohingya population impacted the environment, emotions, and economy of the host population. With the Rohingya population and host-community population living very closely together with easy access between settlements, both populations shared threats of diarrheal disease outbreaks. Any comparison of diverse data that were captured from both the populations was likely to serve as the basis for better understanding of the prevailing differentials in characteristics when compared between the Rohingya refugee population and host-community residents. 

### 2.4. Operational Definitions

We have used the following operational definitions in this study:**Variables****Definitions**DiarrheaAccording to World Health Organization, diarrhea is the passage of loose, watery stools, occurring three or more times in 24 h.Water seal latrineA toilet that has a squatting pan with a water seal with the pit lying immediately below.Pit latrineIt has a squatting pan and simple pit in the ground lying directly below but without any water seal.Tap waterA form of chlorinated public water supply to maximize drinking of safe water, while the water is acceptably free from pathogens that have potential to cause disease and often grow in water supply basins.Shallow tube wellsThose having a depth that is drilled to ≤100 feet, generally easy to install, requiring little effort in maintenance, and giving easy access to drinking water, as they are installed in the neighborhood.Deep tube wellsThose with depths of 500–700 feet, are expensive, difficult and time-consuming to install, and broadly located but give access to relatively safer drinking water.

### 2.5. Data Collection

While implementing the strategy of treating a relatively large number of diarrheal patients, icddr,b felt the responsibility of appraising the activities of the Health Sector, Cox’s Bazar, thereby sharing all relevant information with health systems of the Government of Bangladesh and humanitarian agencies helping the Rohingya refugee population in settlements and neighborhood-residing host-country citizens. The data collection process was carried out to not cause any harm to the respondents, including psychological suffering. The diarrhea patients seeking care from the DTCs, either from the Rohingya population or host-community population, and those hospitalized and who provided an adequate stool specimen for rapid diagnostic test were invited to participate to the study. Relevant data were collected, which included their WASH and ORS use at the household level. Moreover, measurements of nutritional status and collection of fresh stool specimen were carried out. All these activities were initiated after obtaining voluntary informed written consent from the respondents. In case of children, the consent of their parents and/or guardians was obtained. When they were unable to read, the consent form was read aloud to the participant or his/her guardian/parents. A copy of the consent form was given to the respondent for his/her reference. The consent form was written in simple Bangla language, so it could be easily followed by the participants, even those with little or no formal schooling. When assistance was needed, DTC staff members hailing from the DTC’s locality and who are familiar with the dialect of the Rohingya refugee population were sought for better communication with them, particularly during interviewing process. In the case of participants 11–17 years of age, in addition to their assent, consent of their parents and/or guardians was also obtained. The staff members clearly mentioned to the participants that answering the questions would not cause any risk to him/her or his/her family, and there would be no direct benefit to him/her or his/her child after responding to the questionnaire. Moreover, their participation in the study might serve as a basis for an intervention program among the Rohingya refugee population and host population that would benefit him/her and others in the community by implementing newer or better health care services. Participants were informed that they or their family members would not get any remuneration for participation, and they would not have to pay any compensation for participating in the study. They were clearly informed about their rights to cease participation at any point of the interviewing process as well as the study. All safety measures were taken to keep participants’ information confidential. The individual data of the participants are stored in locked cabinets and password-protected computer files, and only key researchers have access to that information. The dataset contains the name and address of the participants, but that information was not used during the data analysis, writing the report, or the manuscript. Participants were kept informed that they would be visited at their household level once a month for three consecutive months to know their health status as well as for participation in community outreach health promotional activities. Field research assistants involved in data collection following standard operating procedure (SOP) had adequate schooling and skills in performing their assigned tasks. Their activities were overseen time to time by one experienced supervisor. Any detected anomalies in the collected data were reviewed and resolved immediately [33,34,35].

### 2.6. Statistical Analysis

WASH variables were our explanatory variables of interest. Outcome variables included increasing years of provision of service deliveries. STATA (version 15.0 IC, College Station, TX, USA: StataCorp LLC) was used for data analysis. Analyses consisted of descriptive as well as analytical methods. Findings concerning WASH variables of interest were compared year-wise between three different consecutive years (year 1 comprised April 2018 to March 2019; year 2 was from April 2019 to March 2020; and year 3 included April 2020–March 2021). Data visualization was accomplished by commonly used plotting, such as bar diagram. Descriptive statistics aided in summarizing data that also included frequency and proportion for categorical variables. The chi-square (χ^2^) test for trend was computed when one variable is binary, and the other is ordered and categorical, and an attempt was made to assess whether the association between two variables followed a significant trend. To identify the characteristics associated with the Rohingya refugee population hospitalized with diarrhea episodes, simple and multiple logistic regression analysis was performed. Strength of association was expressed as odds ratios (ORs) with their 95% confidence intervals (CIs). Variables with a *p*-value less than 0.2 in the bivariate models were initially considered for multivariable logistic regression modeling [36]. Only the significant variables were retained in the final model. A *p*-value less than 0.05 was considered statistically significant. 

## 3. Results

Among the Rohingya population, public tap water practice was 38.5% (*n* = 331/860) in year 1, which increased to 84.7% (*n* = 392/463) in year 2 and 91% (*n* = 244/268) in year 3, χ^2^ for trend = 11.78, *p* < 0.001. Deep tube well water use was 31.3% in year 1, changed to 8.0% in year 2, and was 8.2% in year 3, χ^2^ for trend = 82.05, *p* < 0.001. Shallow tube well water practice was 25.8% in year 1, shifted to 3.7% in year 2, and was 0.4% in year 3; χ^2^ for trend = 123.05, *p* < 0.001 (Table 2).

Household members using a water seal latrine was 13.3% in year 1, increased to 21.4% in year 2, and was 31.7% in year 3, χ^2^ for trend = 15.87, *p* < 0.001. Use of a pit latrine without water seal was 82.8% in year 1, changed to 78.2% in year 2, and was 68.3% in year 3, χ^2^ for trend = 0.74, *p*-value 0.391 (Table 3).

**Figure 3:**
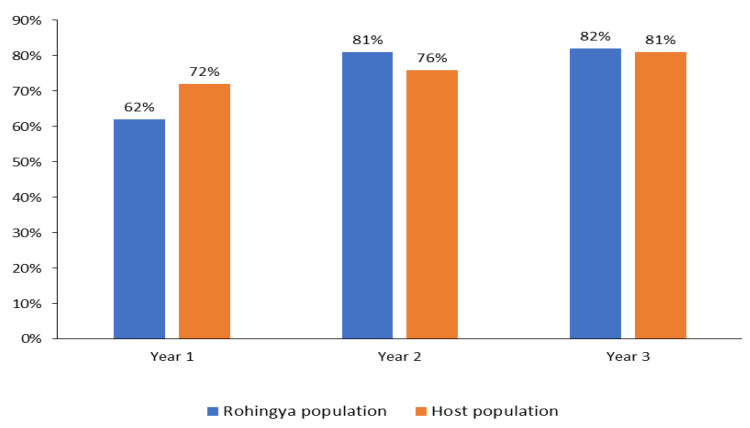
Changing trend of ORS use at home before attending DTCs by the hospitalized Rohingya population and neighborhood host-community population over three years in Teknaf and Ukhia sub-districts, Cox’s Bazar, Bangladesh 2018–2021. Year 1: from 22.04.18 to 31.03.19; year 2: from 01.04.19 to 31.03.20; year 3: from 01.04.20 to 31.03.21.

Use of ORS at home by the hospitalized Rohingya refugee population before seeking care from DTCs changed from 61.5% (529/860) in year 1 to 81% (375/463) in year 2 and to 82.1% (220/268) in year 3. For the host-community hospitalized population, ORS use at home before reporting to DTCs changed from 71.6% (1071/1495) in year 1 to 75.7% (888/1173) in year 2 and to 81.4% (237/291) in year 3; χ^2^ for trend = 16.10, *p* < 0.001 (Figure 3).

After simultaneous adjustments for covariate handwashing before food preparation in the logistic regression model, age of the Rohingya refugee population care seekers from DTCs, age of 5–14 years (AOR 2.37; 95% CI 1.96–2.85; *p* < 0.001), age of 15 years and more (AOR 2.39; 95% CI 1.64–3.50; *p* < 0.001), drinking non-tube well water (AOR 0.42; 95% CI 0.36–0.50; *p* < 0.001), soap use after using toilet (AOR 2.19; 95% CI 1.47–3.28; *p* < 0.001), use of non-sanitary toilet facility (AOR 0.49; 95% CI 0.41–0.58; *p* < 0.001), father’s lack of schooling (AOR 0.58; 95% CI 0.48–0.70; *p* < 0.001), mother’s lack of schooling (AOR 0.13; 95% CI 0.11–0.16; *p* < 0.001), some dehydration (AOR 0.44; 95% CI 0.31–0.62; *p* < 0.001), and severe dehydration (AOR 0.52; 95% CI 0.36–0.74; *p* < 0.001) were significantly associated with the Rohingya refugee population enrolled into the diarrheal disease surveillance (Table 4). 

Adjusted OR from a multivariable model included age of the respondent, drinking non-tube well water, use of no soap after using the toilet, use of non-sanitary toilet facility, father’s lack of schooling, mother’s lack of schooling, some dehydration, and severe dehydration. 

Reference categories included age of 0–4 years, drinking tube well water, use of soap after using toilet, use of sanitary toilet facility, father’s schooling, mother’s schooling, and no dehydration. 

Variance inflation factor (VIF) examined the multicollinearity status between independent variables before performing logistic regression analysis. The VIF values were observed to be less than 2.0. 

## 4. Discussion

The sudden arrival of the Rohingya population in large numbers led to creating spontaneous makeshift settlements in open lands, mostly vacant, low-lying, non-agriculture fields as well as steep hillsides, causing environmental deprivation including loss of fast-growing, large forest areas. The vulnerability of the Rohingya refugee population further amplified soon during seasonal environmental events, including monsoon, heavy showers, and flash floods. Those, in turn, threatened to enhance the Rohingya population’s vulnerability to outbreaks of a wide range of infectious illnesses, including AWD, cholera, and shigellosis. The government of Bangladesh along with international humanitarian agencies and NGOs provided support including relief, shelters, food, clean drinking water, medical care, and WASH services. It is well-known that particularly in emergencies and crisis settings, high levels of childhood malnutrition, worsening sanitary conditions, and repeated infectious diseases with inadequate health service delivery in settlements are common and are potentially life-threatening, predominantly in the case of disadvantaged children [20,23,37,38,39,40,41,42,43,44]. 

Clean drinking water, hygiene, and sanitation are observed to play important roles in maintaining optimal health. However, such clean drinking water and good sanitation would not prevent the occurrence of infectious diseases in the absence of good hygiene practice. In humanitarian crisis settings, there is an immediate need for strategy formulation in WASH-related risk analysis of abrupt and overdue health impacts, followed by development and implementation of participatory inventory strategies and adoption of measures targeting reduction of WASH-related risks. A recent multi-country, mixed-methods evaluation indicated that the most effective disease-risk reduction in humanitarian settings following implementation of interventions was likely to achieve its maximum only when observed ranges of effectiveness are narrow instead of widely dispersed, and appropriate attentions are given to the implementation factors [45]. To put the study into a larger context, the strategy must emphasize the existing evidence to strengthen policy and practice. Additionally, the strategy must underscore the need for concomitant strengthening of the evidence base. The Rohingya refugee population shelters and community facilities are still in the process of rapid up-gradation. Settlements have been developed over the months as stronger and safer, including better roads with electric lighting during night hours, water and sewerage drainage, culverts and bridges, walkways, as well as forest and hill preservation [46,47,48,49,50,51]. 

The vast majority of the Rohingya population are depending on solar power for lighting their homes, getting drinking water from water pumps, and filtering available water, and there is the presence of power round-the-clock in diverse health facilities. All these are visible examples of gradual improvements in the living standards of the Rohingya refugee population dwelling in the settlements [52]. 

The standard water seal latrine design provided to the Rohingya population in settlements is often called a pour-flush pit latrine. The water seal prevents odor and insects from entering the latrine from the pit lying immediately below. Water is thrown into the pan, and it gets washed down with the excreta through the water seal. Another type of latrine is known as a pit latrine, which facilitates infiltration of liquids into the ground, and it acts as a device for storage and later treatment of excreta. Humanitarian aid agencies have installed deep, machine-drilled boreholes throughout the settlements to provide safe drinking water supplies in the settlements. These endeavors are in the form of centralized chlorinated public water supplies to the target population to maximize safe drinking water distribution, thereby ensuring access to safer water free from pathogens. These enteric pathogens are known to have diarrhoeagenic potentials and commonly grow in water supply reservoirs. Aid agencies working in settlements for the Rohingya refugee population generally use automatic online dosing chlorination for water treatment. This treatment method involves introducing a measured amount of chlorine solution into the water pipeline from the borehole source through a dosing pump, followed by permitting adequate retention time in the water-storage tank to allow the process of disinfection. Pipeline distribution networks to public tap stands allow access and collection of water from neighborhoods in reasonable quantities and with acceptable quality and are essential in settlements, as the Rohingya population is at risk for outbreaks of waterborne diseases including cholera and shigellosis. Tube wells are termed boreholes or water wells. It is a device installed to collect groundwater. However, different types of tube wells are used for groundwater collection in Bangladesh, including the settlements of Cox’s Bazar. Shallow tube well water is more likely to be contaminated than deep tube well water. Chlorine tablets and safe water collection and storage containers were distributed, and training for their use was given to the Rohingya population. Increasing numbers of Rohingya refugee population families using piped water distribution systems for accessing chlorinated water has been reported, while the others are collecting their water for domestic purposes from tube wells. 

The WASH program in settlements and host communities aims to ensure reliable, adequate, rational, and decent access to safe water for drinking and domestic needs. Drinking-water surveillance at community point sources is ongoing in Rohingya population settlements as a vigilant public health assessment that steadily monitors the safety of supplied water. According to water-quality surveillance (WQS) in the Rohingya refugee population settlements (January–April 2021), water testing results indicated that 93% (*n* = 156), 4% (*n* = 6), 2% (*n* = 4), and 1% (*n* = 1) had *E. coli* contamination: 0 cfu/100 mL (according to WHO guideline value and Bangladesh standard), 1–10 cfu/100 mL (intermediate risk), 11–99 cfu/100 mL (high risk), and >100 cfu/100 mL (very high risk), respectively [53]. The pipeline water system was observed to supply safe water in terms of *E. coli* count from its source. However, regular monitoring and reporting are essential to apprehend any risks related to contamination.

To meet the huge demand for water made by the Rohingya refugee population living in different settlements, the use of surface water has been considered as one of the reasonable options to reduce the intensity of groundwater crisis in selected areas. A surface water-treatment plant is known to be a centralized system that can monitor and control water quality and quantity. These plant-operated water supplies are subjected to a different type of tests, such as turbidity, total dissolved solids, free residual chlorine, pH, and electrical conductivity. Surface water is treated by batch alum sedimentation and chlorination process. After water testing, safe chlorinated water is pumped to gravity tanks that are connected by standpoints for distribution to communities, mostly uphill. The water level of each surface water reservoir is monitored by measuring with meter gauge. Regular recordings of data help in predicting water storage and water-level reduction rate particularly in dry season because of evaporation, loss due to seepage, and other environmental parameters. Such evidence-based information is essential to set strategies to overcome water crisis [54,55].

Additionally, to promote optimal hygiene practices, the WASH program is providing WASH supplies such as soap, menstrual hygiene products, water storage containers, etc., to the Rohingya population. Aid agencies, through regular visits, ensure steady use and proper maintenance of handwashing stations in places of gathering [11,12,56,57,58]. 

Due to the continuing arrival of very large numbers of the Rohingya population and their increase population density, the living conditions of the Rohingya refugee population were challenging, with the influx continuing for several months altogether. The initial strategy of the humanitarian agencies focused on the emergency provision of water and latrines and the distribution of hygiene materials. Gradually, aid agencies moved to the next phase, with emphasis on standard designs for improved construction of water points, semi-permanent toilets, support for regular operation, and maintenance of those toilets, including fecal sludge treatment, with more focusing on hygiene behavior change, wide-scale community engagement, and solid waste disposal. Reflections of the overall stability of the response from the Rohingya population were observed gradually. The increasing capacity of the aid agencies and their partners was noted, and their intentions to take advantage of their ability to engage the communities in a more meaningful way were also reported. Thus, it is expected that community engagement throughout the implementation of the strategy will expand further, and gradually, more emphasis on hygiene will be noted as an important key behavior [59].

There are indications of the steadiness of these scenarios in settlements, which have been validated by the present study, indicating significant improvements in WASH service delivery platforms. Those have been observed after reviewing the increasing trends in accessing WASH facilities by the Rohingya refugee population.

The study observed an increasing trend in ORS use patterns by the AWD-sick individuals at household level living in the Rohingya population camps and nearby host communities. Capturing relevant data from a fairly large number of hospitalized AWD patients during round-the-clock service delivery by a network of DTCs serving both the Rohingya population and host communities were the strengths of the study. Humanitarian agencies are continuing their service deliveries in settlements in collaboration with relevant departments of the government of Bangladesh. These include supply of safe water for drinking, cooking, and personal hygiene. Those agencies are providing serviceable toilets of optimal standards. The Rohingya population is receiving supplies of soap bars for personal and household hygiene maintenance as well as laundry every month. As a result of increasing access to piped water supply, the Rohingya refugee population is less frequently accessing water from tube wells. Women and girls of reproductive age are receiving menstrual hygiene management kits. Those agencies without any disruption are providing safe sanitation support such as maintenance of latrines and operation of small-scale sewage-treatment plants. They are maintaining bathing spaces, too. Additionally, they are enabling people with disabilities in accessing WASH platforms. To further strengthen WASH services in line with the COVID-19 pandemic, those involved in infection prevention and control (IPC) measures are actively accomplishing regular disinfection of water points, tap stands, water reservoir tanks, tube wells and their platforms, as well as latrines and bathing facilities in camps. Similar activities are also going on in host communities [12,59,60].

To provide or improve access to potable water by the both the Rohingya refugee population and host-community population during the safe water crisis period, strategies can be formulated for temporary potable water transport, storage, and distribution, particularly targeting densely populated and high-demanding areas. Larger containers could be distributed to families in order to facilitate storage of a higher quantity of water at the family level. Moreover, in such settings, bulk water chlorination may be the best means of quickly providing large amounts of safe potable water to the target population. 

The study is not without limitations. Of them, an important one is that this study included only those who were hospitalized in the DTCs. Thus, the study is lacking relevant information from those who sought out-patient care with apparently less severe diseases. Community cases and those not reporting to DTCs were also not studied. Accordingly, the study observations may not be generalizable. Moreover, data were captured by interviewing, which may have been subjected to recall bias of the respondents. The study lacks information on the intake of volume of ORS as well as hours or days of ORS use at home before seeking care from DTCs. However, the present study captured information from a fairly sufficient number of cases who were hospitalized in widely distributed DTCs from the Rohingya refugee population shelters as well as Bangladeshi communities, thus comprising the appreciating strength of the study. 

## 5. Conclusions

Humanitarian agencies and their local-level partners must regularly monitor sanitation facilities, such as disposal of greywater and contents from septic tanks and cesspools. More and more use of water seal toilets should be promoted at a relatively higher pace. Monitoring of relevant performance of the local partner workforce should be observed regarding whether they are complying with the procedures for desludging and ensuring safe disposal of excreta. When necessary, repairs are to be made on time and targeted sanitation and necessary improvements completed as and when the situation demands. Humanitarian agencies must be actively involved in regular monitoring of the drinking water sources, particularly free residual chlorine levels of water points as well as regular monitoring of water quality at household level. Organization of appropriate remedial actions should be taken if necessary. In case of any acute water crisis, especially during the dry season, excess needs of the Rohingya population may be met by potable water trucking as long as the crisis persists. Handwashing with soap practices can be reinforced through the promotion and provision of soap and installation of handwashing stations in public health places, including restaurants and markets. A quick response to diarrhea outbreaks to control spread can be achieved by identification of affected water sources, instituting corrective measures, and optimizing information sharing as a helpful resource. Provision and maintenance of handwashing stations, such as ensuring regular availability of soap in schools, markets or other public spaces, food shops, and other relevant settings, should be expanded in newer places, and existing services should be further reinforced. Substantial improvements in household-level use of ORS before hospitalization in DTCs can replicate the increasing awareness and knowledge. Additionally, easy access to packaged ORS at the household or community level as a result of promotional use of ORS outreach activities in settlements is noteworthy. Moreover, it reflects that aid agencies are enhancing motivation through repeated home visits, and also health education is imparted during those visits with an emphasis on starting ORS as soon as diarrhea begins, thereby eliminating the trip to the DTCs.

## Figures and Tables

**Table 1:** Description and capacities of the DTCs.

Name and Location of DTC	Health Facility Code	Starting Date	Ending Date	Capacity
Leda DTC	HF: 197	10 April 2018	31 March 2019	14 beds
Leda DTC (2nd Time)	HF: 197	8 October 2019	31 January 2020	14 beds
Leda DTC (3rd Time)	HF: 197	1 May 2020	Continuing till now	14 beds
Teknaf DTC	HF: 567	7 August 2018	30 April 2020	30 beds
Shamlyapur DTC	HF: 585	17 May 2018	31 March 2019	20 beds
Balukhali DTC	HF: 182	2 May 2018	31 December 2018	6 beds
Ukhia DTC	HF: 312	1 June 2018	31 December 2018	30 beds

**Table 2:** Changing trend showing water source used by the Rohingya population and neighborhood host-country population over three years in the Teknaf and Ukhia sub-districts, Cox’s Bazar, Bangladesh 2018–2021.

	Year 1		Year 2		Year 3	
Indicator	*n* = 2355 (%)		*n* = 1636 (%)		*n* = 559 (%)	
Water source	Rohingya population *n* = 860 (%)	Host population *n* = 1495 (%)	Rohingya population *n* = 463 (%)	Host population *n* = 1173 (%)	Rohingya population *n* = 268 (%)	Host population *n* = 291 (%)
Public tap	331 (38.5)	46 (3.1)	392 (84.7)	29 (2.5)	244 (91.0)	12 (4.1)
Deep tube well	269 (31.3)	638 (42.7)	37 (8.0)	445 (37.9)	22 (8.2)	187 (64.3)
Shallow tube well	222 (25.8)	699 (46.8)	17 (3.7)	580 (49.4)	1 (0.4)	49 (16.8)
Other	38 (4.4)	112 (7.5)	17 (3.7)	119 (10.1)	1 (0.4)	43 (14.8)

Year 1: from 22.04.18 to 31.03.19; year 2: from 01.04.19 to 31.03.20; year 3: from 01.04.20 to 31.03.21.

**Table 3:** Changing trend of use of pit latrine with water seal and without water seal by the Rohingya population and neighborhood host-country population over three years in Teknaf and Ukhia sub-districts, Cox’s Bazar, Bangladesh 2018–2021.

	Year 1		Year 2		Year 3	
Indicator	*n* = 2355 (%)		*n* = 1636 (%)		*n* = 559 (%)	
Type of toilet	Rohingya population *n* = 860 (%)	Host population *n* = 1495 (%)	Rohingya population *n* = 463 (%)	Host population *n* = 1173 (%)	Rohingya population *n* = 268 (%)	Host population *n* = 291 (%)
Pit latrine, slab with water seal	114 (13.3)	376 (25.2)	99 (21.4)	315 (26.9)	85 (31.7)	128 (44.0)
Pit latrine, slab without water seal	712 (82.8)	814 (54.4)	362 (78.2)	664 (56.6)	183 (68.3)	160 (55.0)

Year 1: from 22.04.18 to 31.03.19; year 2: from 01.04.19 to 31.03.20; year 3: from 01.04.20 to 31.03.21.

**Table 4:** Characteristics associated with the enrolled Rohingya refugee population compared to the enrolled neighborhood host-country population over three years in Teknaf and Ukhia sub-districts, Cox’s Bazar, Bangladesh 2018–2021.

Characteristics	AOR	95% CI	*p*
Age			
5–14 years	2.37	1.96–2.85	<0.001
15+ years	2.39	1.64–3.50	<0.001
Drinking non-tube well water	0.42	0.36–0.50	<0.001
Use of no soap after using the toilet	2.19	1.47–3.28	<0.001
Use of non-sanitary toilet facility	0.49	0.41–0.58	<0.001
Father’s lack of schooling	0.58	0.48–0.70	<0.001
Mother’s lack of schooling	0.13	0.11–0.16	<0.001
Some dehydration	0.44	0.31–0.62	<0.001
Severe dehydration	0.52	0.36–0.74	<0.001

Abbreviations: AOR, adjusted odds ratio; CI, confidence interval.

## Data Availability

This dataset and materials are available via the corresponding author and can be accessed on a valid request only.

## Abbreviations

WASH	Water, sanitation and hygiene
DTCs	Diarrhea-treatment centers
ORS	Oral rehydration solutions
*E. coli*	*Escherichia coli*
icddr,b	International Centre for Diarrhoeal Disease Research, Bangladesh
UNICEF	United Nations Children’s Fund
NGO	Non-governmental organization
OCV	Oral cholera vaccine
EWARS	Early warning and alert response system
DDSS	Diarrheal Disease Surveillance System
WQS	Water-quality surveillance
